# The impact of red blood cell storage duration on clinical outcomes in pediatric cardiac surgery: a systematic review and meta-analysis

**DOI:** 10.3389/fped.2025.1649610

**Published:** 2025-12-16

**Authors:** Xing Jin, Qingyu Zhang, Ye Sun, Zhiting Dong, Wenzhe Jin

**Affiliations:** 1 Department of Pain, Yanbian University Hospital, Yanji, Jilin, China; 2 Department of Anatomy, School of Medicine, Yanbian University, Yanji, Jilin, China; 3 Department of General Surgery II, Affiliated Hospital of Beihua University, Jilin City, Jilin, China

**Keywords:** red blood cell storage lesion, pediatric cardiac surgery, cardiopulmonary bypass, blood transfusion, meta-analysis, perioperative outcomes

## Abstract

**Background:**

The impact of red blood cell (RBC) storage duration during cardiopulmonary bypass (CPB) priming in pediatric cardiac surgery remains unclear.

**Objective:**

To evaluate whether RBC storage time affects perioperative outcomes in children undergoing cardiac surgery.

**Methods:**

We performed a systematic review and meta-analysis of studies comparing fresh vs. longer-stored RBCs for CPB priming in pediatric patients. Databases searched included PubMed, EMBASE, Cochrane Library, and Web of Science (through May 2025). Primary outcomes were mortality, infection/sepsis, respiratory complications, and multiple organ dysfunction syndrome (MODS); secondary outcomes included mechanical ventilation duration, ICU stay, and intraoperative lactate levels.

**Results:**

Ten studies (including one randomized controlled trial) were included. No significant differences were found between groups in any primary or secondary outcomes, except for a slightly shorter ICU stay in the fresh RBC group (mean difference = –1.08 days), with high heterogeneity.

**Conclusions:**

RBCs stored within standard durations appear safe for CPB priming in pediatric cardiac surgery. These findings support current transfusion practices and underscore the need for further high-quality randomized trials.

**Systematic Review Registration:**

https://www.crd.york.ac.uk/prospero/display_record.php?RecordID=1015198, PROSPERO CRD420251015198.

## 1 Introduction

In pediatric cardiac surgery, the priming volume required for cardiopulmonary bypass (CPB) circuits typically ranges from 300 to 500 mL, often exceeding 50% of the total blood volume in infants and young children—markedly higher than the approximate 10% observed in adult surgeries (1, 2). This substantial difference renders red blood cell (RBC) transfusion an indispensable component in the initiation of CPB (3, 4). Given the limited circulating blood volume in infants (∼80 mL/kg) and their high oxygen consumption rate (6–8 mL/kg/min), both the quality and volume of transfused RBCs are critical determinants for maintaining perioperative oxygen delivery and organ perfusion stability (5, 6).

However, during storage, RBCs undergo progressive “storage lesions,” including decreased membrane stability, depletion of ATP and 2,3-diphosphoglycerate (2,3-DPG), and the release of microparticles and pro-inflammatory cytokines (7–9). These pathological changes may compromise oxygen delivery efficiency and exacerbate intra- and postoperative complications when transfused into pediatric patients (10–12). Although current FDA and European guidelines permit the use of RBCs stored for up to 35–42 days, whether all storage durations within this window are equally safe remains a topic of debate (13, 14).

Several large randomized controlled trials (RCTs), such as RECESS (15) and ABLE (16), have demonstrated that RBC storage duration does not significantly impact clinical outcomes in adults. Similarly, the ABC PICU trial found no significant difference in the incidence of multiple organ dysfunction syndrome (MODS) between fresh (≤7 days) and standard-issue RBCs in critically ill pediatric patients (17). However, caution should be exercised when extrapolating these findings to pediatric cardiac surgical populations due to key physiological differences: (1) the RBC volume used for CPB priming greatly exceeds that of conventional transfusions (18); (2) children with congenital heart disease often have immature circulatory and immune systems (19); and (3) developing organs—particularly the brain—are more susceptible to ischemia-reperfusion injury (20).

In recent years, increasing attention has been paid to the relationship between RBC storage duration and postoperative outcomes in pediatric cardiac surgery. Some observational studies have reported associations between longer storage durations and increased risks of postoperative infections (21, 22), pulmonary and renal complications (22), prolonged mechanical ventilation, and enhanced inflammatory responses (23). However, other studies have found no significant associations between storage duration and major outcomes, resulting in substantial variability in clinical practice.

Although the 2016 AABB guidelines state that RBCs of any storage duration within the permissible shelf life are acceptable for neonatal and pediatric transfusion (24), many institutions still preferentially use fresher RBCs for CPB priming—decisions often based on theoretical concerns or institutional habits rather than robust evidence (23).

Given the absence of specific consensus guidelines regarding RBC storage thresholds in pediatric CPB settings, and the lack of clear recommendations in current transfusion protocols, clinical decisions largely depend on institutional experience. This underscores the urgent need to systematically review the available evidence to determine whether RBC storage duration significantly affects outcomes in pediatric cardiac surgery.

Therefore, this study aims to perform a systematic review and meta-analysis to evaluate the impact of RBC storage duration on major outcomes (including mortality, infection, and MODS) and secondary outcomes [such as mechanical ventilation duration, Intensive Care Unit (ICU) length of stay, and intraoperative lactate levels] in children undergoing cardiac surgery with CPB. The goal is to establish a more evidence-based transfusion window for this vulnerable population and to inform optimization of perioperative blood management strategies.

## 2 Methods

### 2.1 Study design and registration

This systematic review and meta-analysis was conducted to evaluate the impact of RBC storage duration on clinical outcomes in pediatric cardiac surgery. The study adhered to the PRISMA 2020 (Preferred Reporting Items for Systematic Reviews and Meta-Analyses) guidelines. The study protocol was prospectively registered in the PROSPERO database (Registration No. CRD420251015198). Ethical approval was not required as only publicly available data were used.

### 2.2 Literature search strategy

A comprehensive search was performed in PubMed, EMBASE, the Cochrane Library, and Web of Science from January 2000 to February 2025. Only English-language articles were considered. The search strategy was constructed based on the PICOS framework, with keywords covering the population (e.g., “pediatric cardiac surgery,” “congenital heart disease,” “children,” “infants”), interventions (e.g., “red blood cell storage,” “RBC transfusion,” “CPB priming,” “cardiopulmonary bypass”), and outcomes (e.g., “mortality,” “infection rate,” “postoperative complications”). Boolean operators (AND/OR) were used for logical combinations. The complete PubMed search string is provided in Supplementary Table S1.

In addition, we manually screened the reference lists of included articles and relevant reviews to identify additional studies. Grey literature, including conference abstracts and dissertations, was also searched to minimize publication bias. All retrieved records were managed using EndNote X9, and duplicates were removed using the software's de-duplication function.

### 2.3 Inclusion and exclusion criteria

Studies were eligible for inclusion if they met the following criteria: (1) design: RCTs or observational studies (prospective/retrospective cohorts); (2) population: pediatric patients ≤18 years undergoing cardiac surgery requiring RBC priming for CPB; (3) exposure: comparison of fresher vs. older stored RBCs (using original study definitions, typically ≤7–14 days vs. >7–14 days); (4) outcomes: at least one postoperative endpoint, including mortality, infection/sepsis, respiratory complications, mechanical ventilation duration, ICU stay, lactate levels, or organ complications.

Exclusion criteria consisted of: (1) adult-only populations; (2) studies unrelated to CPB or RBC storage duration; (3) unextractable or missing data on exposure or outcomes; (4) non-original articles (reviews, case reports); (5) inaccessible full text; and (6) studies combining blood products without separate analysis of RBCs.

Because definitions of “fresh” RBCs varied markedly (≤4 to ≤15 days), an *a priori* threshold-standardized comparison (≤7 vs. >7 days) was prespecified to enhance comparability across heterogeneous definitions.

### 2.4 Data extraction

Two reviewers independently screened titles, abstracts, and full texts and extracted data using a standardized form. Extracted variables included: (1) study characteristics (author, year, country, design); (2) patient demographics (age, sample size, diagnosis); (3) perioperative details (surgical type, CPB priming protocols, transfusion volumes); (4) definitions of RBC storage duration; (5) all reported outcomes.

For continuous variables reported as medians with interquartile ranges, means and standard deviations were estimated using the method of Wan et al. (25). Graph-only data were digitized with GraphPad Prism, and all graph-derived values underwent dual independent extraction with cross-validation. When numerical data were missing or ambiguous, corresponding authors were contacted, although no additional data were obtained.

### 2.5 Quality assessment

Two reviewers independently assessed study quality. Observational studies were evaluated using the Newcastle–Ottawa Scale (NOS) (26), covering selection, comparability, and outcome domains. RCTs, if present, were assessed using the Cochrane Risk of Bias Tool (27), evaluating random sequence generation, allocation concealment, blinding, and completeness of outcome reporting. Disagreements were resolved by consensus or adjudication by a third reviewer.

### 2.6 Statistical analysis

All analyses were performed in Stata 18.0. For dichotomous outcomes, risk ratios (RRs) with 95% confidence intervals (CIs) were calculated; for continuous outcomes, mean differences (MDs) were used. Heterogeneity was quantified using Cochran's *Q* and the *I*^2^ statistic. A random-effects model (DerSimonian–Laird) was applied when substantial heterogeneity existed (*I*^2^ > 50% or *p* < 0.10); otherwise, fixed-effects models were used (28).

Given the marked heterogeneity in storage-day definitions across studies, several prespecified supplementary analyses were conducted: (1) threshold-standardized subgroup analyses (≤7 vs. >7 days); (2) subgrouping by study design (RCT vs. observational); (3) infection subtype (infection vs. sepsis); (4) respiratory complication severity, classified according to original terminology; vague descriptions (e.g., “respiratory events”) were analyzed separately as mild complications to minimize misclassification bias.

Leave-one-out sensitivity analyses were performed for all primary and secondary outcomes. Because one study used an extremely strict definition of “fresh” RBCs (≤4 days), additional analyses excluding this study were conducted across all major endpoints. To address limitations of the DerSimonian–Laird estimator in heterogeneous or small-sample meta-analyses, restricted maximum likelihood (REML) random-effects models were fitted as robustness checks. Funnel plots and Egger's tests were used to evaluate publication bias when ≥10 studies were available. Meta-regression was not performed because no outcome included the minimum number of studies required for reliable analysis (≥10).

### 2.7 Assessment of evidence quality

The overall quality of evidence was rated using the GRADE (Grading of Recommendations Assessment, Development and Evaluation) framework (29), categorizing evidence as high, moderate, low, or very low quality.

### 2.8 Study limitations

Several limitations should be considered when interpreting this review: (1) Most included studies were observational, limiting causal inference; (2) Definitions of postoperative complications—especially respiratory outcomes—were inconsistent and may introduce classification bias; (3) Conversion of medians and IQRs using Wan's method may introduce estimation error, although sensitivity analyses indicated minimal influence on pooled effects; (4) Definitions of “fresh” RBCs varied widely (≤4 to ≤15 days), and CPB priming protocols were incompletely reported in several cohorts; (5) Many studies had small sample sizes, reducing precision; (6) Although threshold-standardized analyses (≤7 vs. >7 days) were conducted, other clinically relevant subgroupings—such as neonatal vs. infant vs. older children, CPB priming volumes, transfusion thresholds, or study-era effects—could not be assessed due to insufficient stratified reporting; (7) Most outcomes included fewer than 10 studies, preventing reliable assessment of publication bias; therefore, small-study effects cannot be excluded.

## 3 Results

### 3.1 Study selection and characteristics

**Figure 1:**
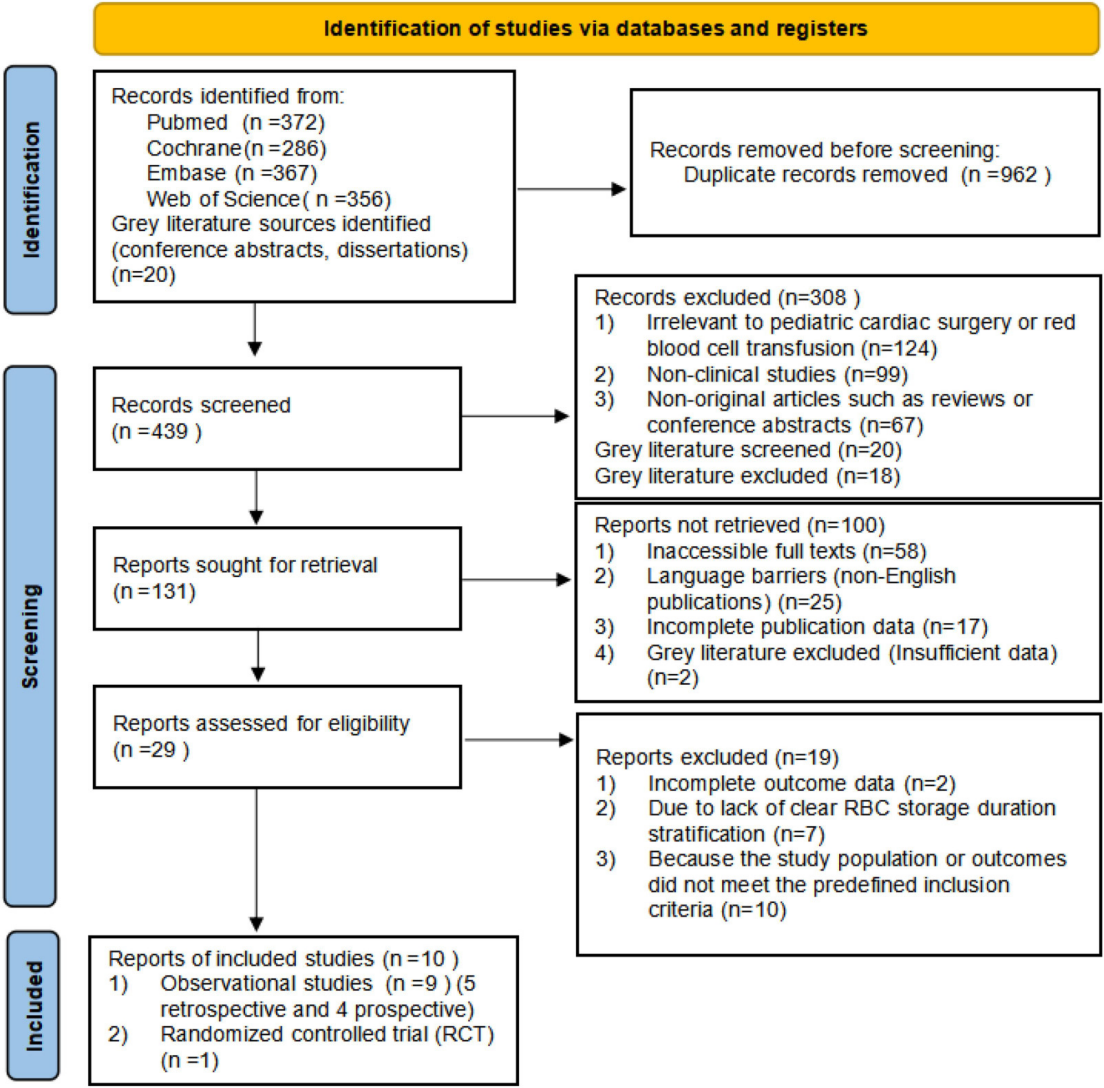
PRISMA flow diagram of study selection process for inclusion in the systematic review and meta-analysis.

A total of 1,381 records were identified through database searches (PubMed, Cochrane Library, EMBASE, and Web of Science). After removal of duplicates, 419 records underwent title and abstract screening. Of these, 129 full-text articles were reviewed, and 10 studies ultimately met the inclusion criteria (Figure 1).

The included studies were published between 2004 and 2023 and comprised 9 observational cohorts (21–23, 30–35)—5 retrospective (22, 23, 30, 31, 34) and 4 prospective (21, 32, 33, 35)—and 1 RCT (36). Sample sizes ranged from 30 to 570, and populations included neonates, infants, and young children (up to 8 years). Definitions of “fresh” RBCs varied considerably, using cut-offs ranging from ≤4 to ≤22 days, typically based on predefined thresholds or median storage durations. The clinical endpoints examined in the included studies encompassed both primary outcomes—postoperative mortality, infection/sepsis, respiratory complications, and MODS—and secondary outcomes, including mechanical ventilation duration, ICU length of stay, and intraoperative lactate levels (Table 1).

**Table 1:** Characteristics of included studies.

No.	First author (Year)	Study design	Sample size (Fresh vs. Stored, *N*)	Age range	Grouping criteria	Outcomes	Key findings
1	Cholette (2015)	Prospective	33 vs. 20 (*N* = 53)	Median 7 months	7–15 days vs. 25–38 days	Infection rate	Higher postoperative infection rate in stored blood group (*p* = 0.004)
2	Ranucci (2009)	Retrospective	116 vs. 76 (*N* = 192)	6.5 vs. 8 months	≤4 days vs. >4 days	Metabolism, complications	Higher incidence of pulmonary and major complications in stored blood group (*p* < 0.05)
3	Redlin (2014)	Retrospective	45 vs. 94 (*N* = 139)	Median 14 days	≤6 days vs. >6 days	CRP, mechanical ventilation	Increased CRP and prolonged ventilation in stored blood group (HR = 1.72)
4	Padiyath (2021)	Retrospective	122 vs. 339 (*N* = 461)	Mean 4.3 years	≤15 days vs. >15 days	Infection, hospital stay	Lower sepsis incidence in stored group (low transfusion subgroup, *p* = 0.0,008)
5	Baltsavias (2014)	Retrospective	118 vs. 452 (*N* = 570)	Median 9 vs. 7 months	≤15 days vs. >15 days	Composite outcomes (death, MODS)	No significant difference in primary/secondary outcomes (*p* > 0.05)
6	Bishnoi (2018)	Prospective	103 vs. 95 (*N* = 198)	4 days–8 years	≤14 days vs. >14 days	Morbidity, metabolism	Only liver dysfunction differed; no significance after adjustment
7	Bishnoi (2017)	Prospective	161 vs. 53 (*N* = 214)	Mean 409 days	≤7 days vs. ≥22 days	Mortality, MOF, metabolism	No significant association between storage time and complications (*p* > 0.05)
8	Schroeder (2005)	Retrospective	12 vs. 8 (*N* = 20)	23 ± 19 vs. 22 ± 16 months	≤12 days vs. >12 days	Lactate level	Higher lactate after CPB in stored group; no difference at surgery end (*p* = 0.0006)
9	Keidan (2004)	Prospective	18 vs. 12 (*N* = 30)	3 days–5 years	≤5 days vs. >5 days	K+, lactate, acid-base balance	Early metabolic differences resolved during/after CPB
10	Martin (2023)	RCT	89 vs. 89 (*N* = 178)	Median 0.6 years	Median 5 days vs. 18 days	MODS, complications	No significant difference in major outcomes between groups (*p* = 0.49)

MODS, Multiple Organ Dysfunction Syndrome; CPB, Cardiopulmonary Bypass; CRP, C-reactive Protein; MOF, Multiple Organ Failure; HR, Hazard Ratio.

### 3.2 Study quality assessment

The 9 observational studies were assessed using the NOS (26), with scores ranging from 5 to 7 (out of 9). Most studies clearly defined study populations and exposure assessment, while approximately half adjusted for confounding factors. However, many studies lacked reporting on follow-up completeness, contributing to lower scores in the outcome domain. Overall, 4 studies were rated as high quality (NOS ≥ 7), and 5 were of moderate quality (score = 5) (Table 2).

**Table 2:** Newcastle–Ottawa scale (NOS) assessment for observational studies.

No.	First Author (Year)	Selection (4 points)	Comparability (2 points)	Outcome (3 points)	Total Score
		1. Clear definition 2. Representativeness 3. Source of controls 4. Ascertainment	5. Adjustment for confounding	6. Objectivity of outcome 7. Adequacy of follow-up 8. Loss to follow-up	
1	Cholette 2015	Yes (1) Yes (1) Yes (1) Yes (1)	Partially adjusted (1)	Yes (1) Yes (1) Not reported (0)	7/9
2	Ranucci 2009	Yes (1) Yes (1) Yes (1) Yes (1)	Not adjusted (0)	Yes (1) Yes (1) Not reported (0)	5/9
3	Redlin 2014	Yes (1) Yes (1) Yes (1) Yes (1)	Partially adjusted (1)	Yes (1) Yes (1) Not reported (0)	7/9
4	Padiyath 2021	Yes (1) Yes (1) Yes (1) Yes (1)	Not adjusted (0)	Yes (1) Yes (1) Not reported (0)	5/9
5	Baltsavias 2014	Yes (1) Yes (1) Yes (1) Yes (1)	Not adjusted (0)	Yes (1) Yes (1) Not reported (0)	5/9
6	Bishnoi 2018	Yes (1) Yes (1) Yes (1) Yes (1)	Partially adjusted (1)	Yes (1) Yes (1) Not reported (0)	7/9
7	Bishnoi 2017	Yes (1) Yes (1) Yes (1) Yes (1)	Partially adjusted (1)	Yes (1) Yes (1) Not reported (0)	7/9
8	Schroeder 2005	Yes (1) Yes (1) Yes (1) Yes (1)	Not adjusted (0)	Yes (1) Yes (1) Not reported (0)	5/9
9	Keidan 2004	Yes (1) Yes (1) Yes (1) Yes (1)	Not adjusted (0)	Yes (1) Yes (1) Not reported (0)	5/9

The single RCT (36) was assessed using the Cochrane Risk of Bias tool (27). It was rated as low risk in terms of random sequence generation, outcome completeness, selective reporting, and other sources of bias, but had unclear risk due to insufficient information on allocation concealment and blinding. The trial was overall judged to have a low risk of bias (Table 3).

**Table 3:** Cochrane risk of bias assessment for RCT.

First author (year)	Random sequence generation	Allocation concealment	Blinding (participants/investigators)	Outcome data completeness	Selective reporting	Other bias	Overall risk of bias
Martin 2023	Low risk (yes)	Unclear	Unclear (double-blinding not specified)	Low risk (dropout rate <5%)	Low risk (outcomes as registered)	None	Low risk

### 3.3 Meta-analysis of primary outcomes

Given the wide variability in definitions of “fresh” RBCs (≤4–≤15 days), all primary outcomes were additionally examined using a standardized ≤7-day threshold to improve comparability. Raw 2 × 2 event counts for all dichotomous outcomes—including mortality, sepsis, infection, MODS, respiratory complications (mild/severe), liver dysfunction, and renal dysfunction—are provided in Supplementary Table S1 to enhance transparency.

#### 3.3.1 Mortality

**Figure 2:**
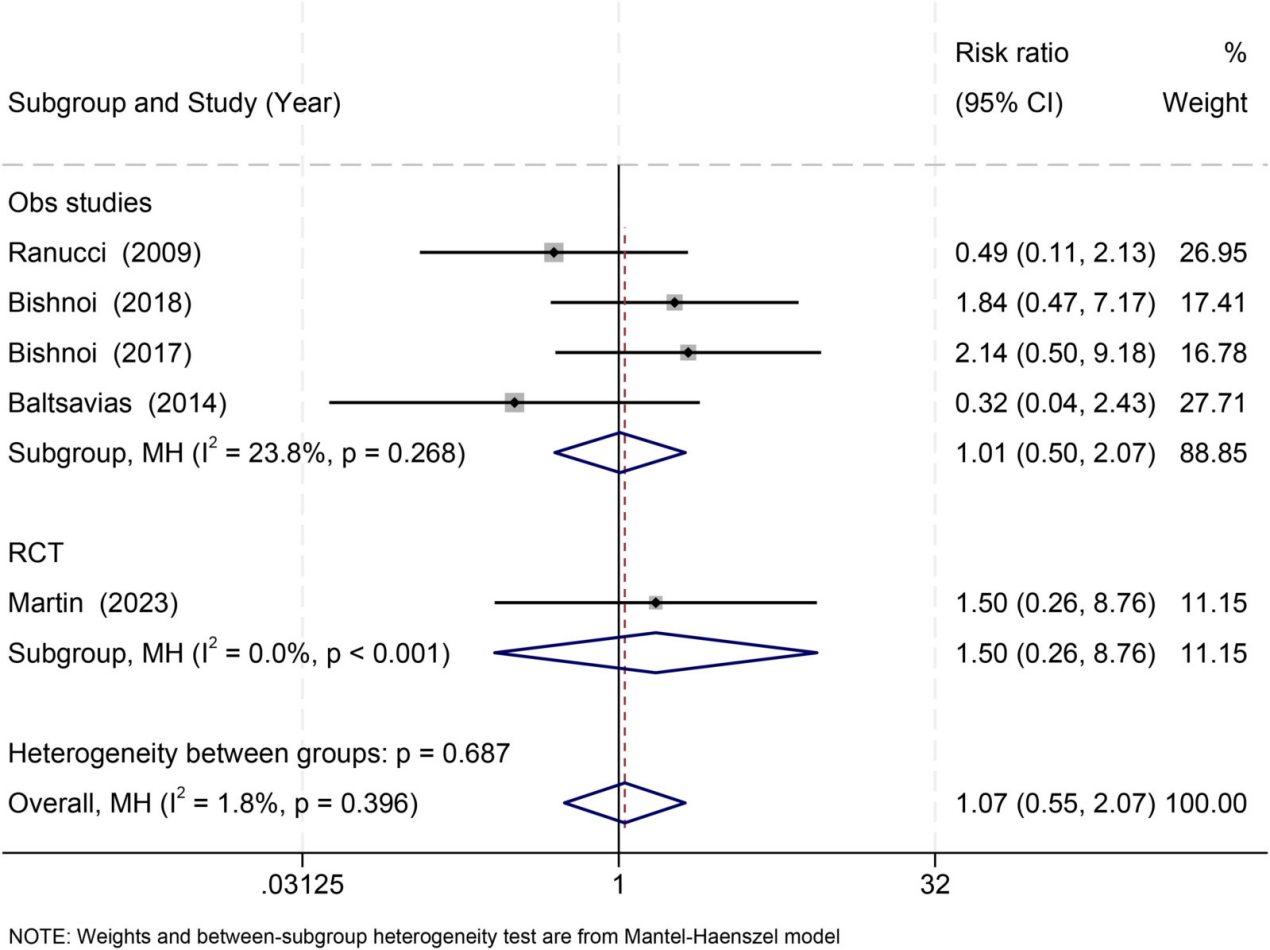
Effect of RBC storage duration on mortality in pediatric cardiac surgery: subgroup and overall estimates.

Five studies [4 observational (22, 31–33) and 1 RCT (36)] reported mortality. Pooled results showed no significant difference between fresh and stored RBCs (RR = 1.07, 95% CI: 0.55–2.07; *I*^2^ = 1.8%) (Figure 2).

To address definitional heterogeneity, a standardized ≤7-day subgroup analysis was conducted. Studies using ≤7 days (22, 33, 36) showed no significant association (RR = 1.20, 95% CI: 0.51–2.82), and studies using >7 days (31, 32) yielded similar findings (RR = 0.91, 95% CI: 0.32–2.60), with no subgroup difference (*p* = 0.686).

Leave-one-out sensitivity analyses demonstrated that no single study influenced pooled estimates, and exclusion of the only study using a ≤4-day threshold [Ranucci 2009 (22)] did not alter results (Supplementary Figures S1, S2).

#### 3.3.2 Infection and sepsis

**Figure 3:**
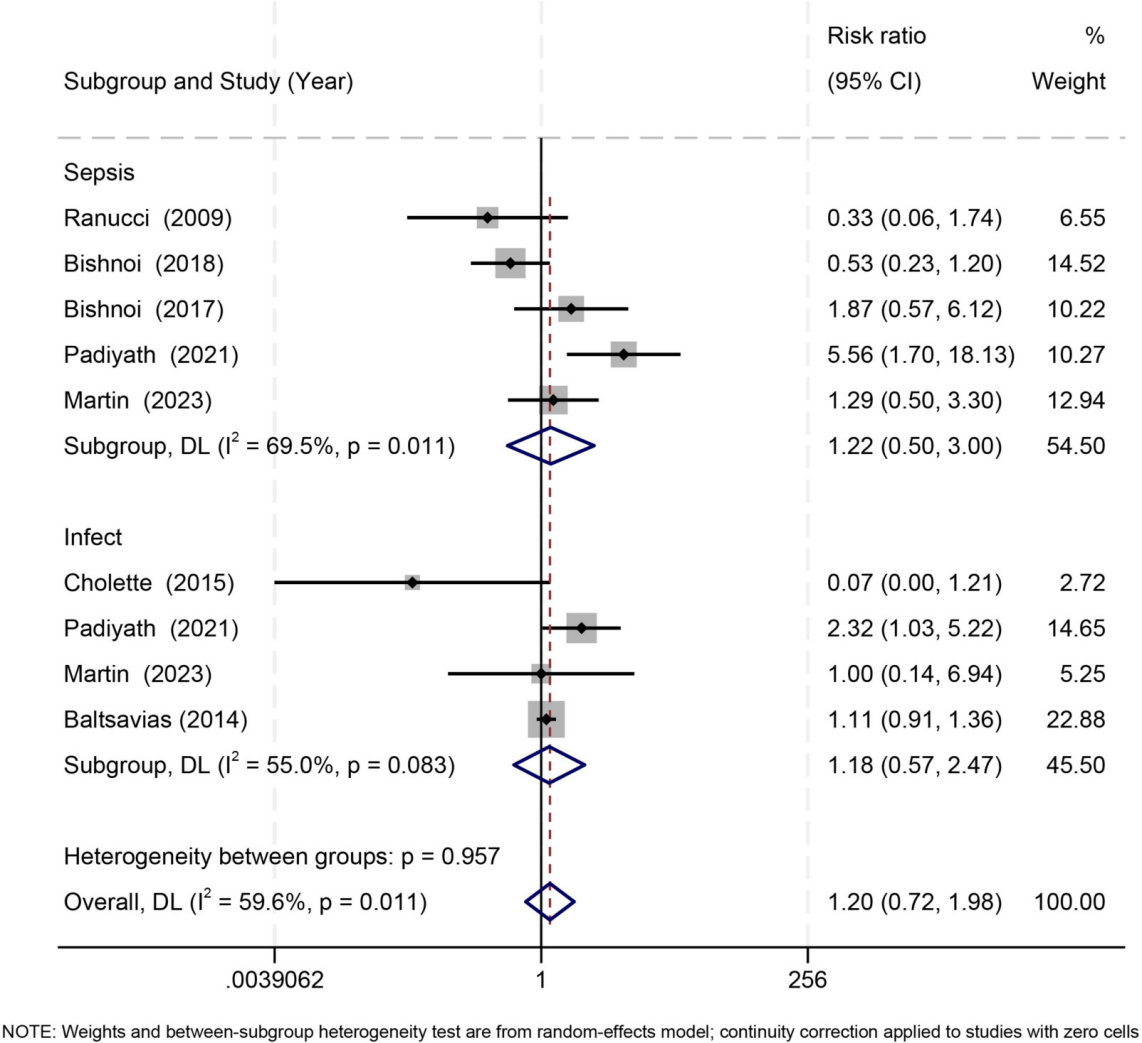
Effect of RBC storage duration on postoperative sepsis and infection in pediatric cardiac surgery: subgroup and overall estimates.

Seven studies (21, 22, 30–33, 36) reported postoperative infection outcomes. Overall, RBC storage duration was not associated with infection risk (RR = 1.20, 95% CI: 0.72–1.98; *I*^2^ = 59.6%). Both sepsis (RR = 1.22, 95% CI: 0.50–3.00; *I*^2^ = 69.5%) and general infections (RR = 1.18, 95% CI: 0.47–2.97; *I*^2^ = 55.0%) showed nonsignificant effects, with no subgroup difference (*p* = 0.957) (Figure 3).

A standardized ≤7-day analysis again demonstrated no significant association for either sepsis or infection, and subgroup differences were absent (all *p* > 0.20) (Supplementary Figures S3, S4).

Sensitivity analyses confirmed stability, and exclusion of Ranucci 2009 (22) did not alter the direction or magnitude of pooled effects (Supplementary Figure S5).

#### 3.3.3 Respiratory complications

**Figure 4:**
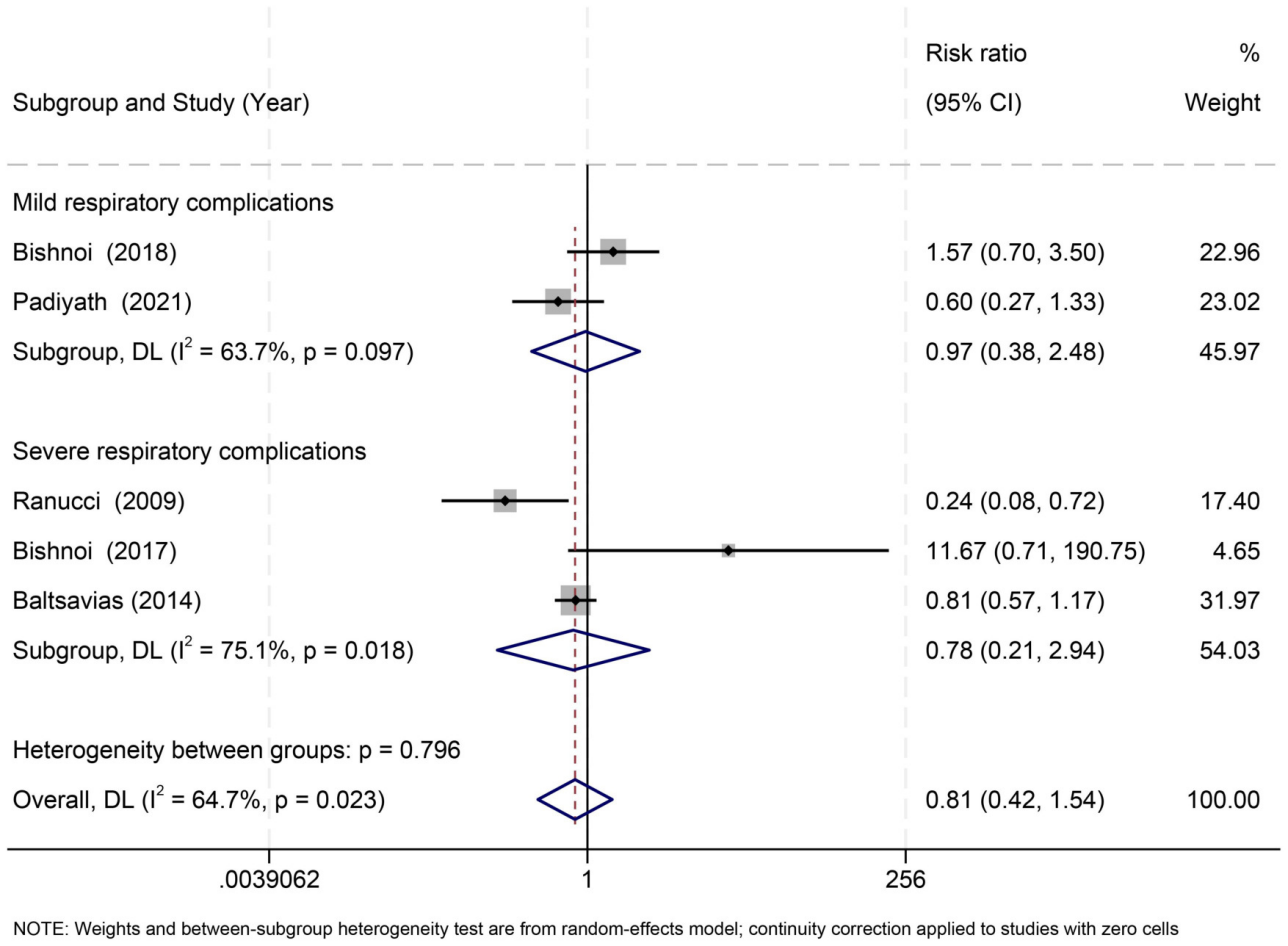
Effect of RBC storage duration on respiratory complications in pediatric cardiac surgery: subgroup and overall estimates.

Five studies (22, 30–33) reported respiratory complications, categorized as mild (nonspecific pulmonary events) (30, 32) or severe (pneumonia, ARDS, respiratory failure) (22, 31, 33). No significant differences were found for mild (RR = 0.97, 95% CI: 0.38–2.48; *I*^2^ = 63.7%) or severe complications (RR = 0.78, 95% CI: 0.21–2.94; *I*^2^ = 75.1%), and overall pooled effects were nonsignificant (RR = 0.81, 95% CI: 0.42–1.54; *I*^2^ = 64.7%) (Figure 4).

Due to definitional heterogeneity, a ≤ 7-day subgroup analysis was performed for severe complications. Results remained nonsignificant in both ≤7-day (22, 33) and >7-day (31) strata (*p* = 0.798) (Supplementary Figure S6).

A sensitivity analysis excluding studies with vague respiratory reporting (those contributing only mild events) did not alter estimates. Leave-one-out analyses—including exclusion of Ranucci 2009 (22)—did not materially affect pooled effects (Supplementary Figure S7).

#### 3.3.4 MODS

**Figure 5:**
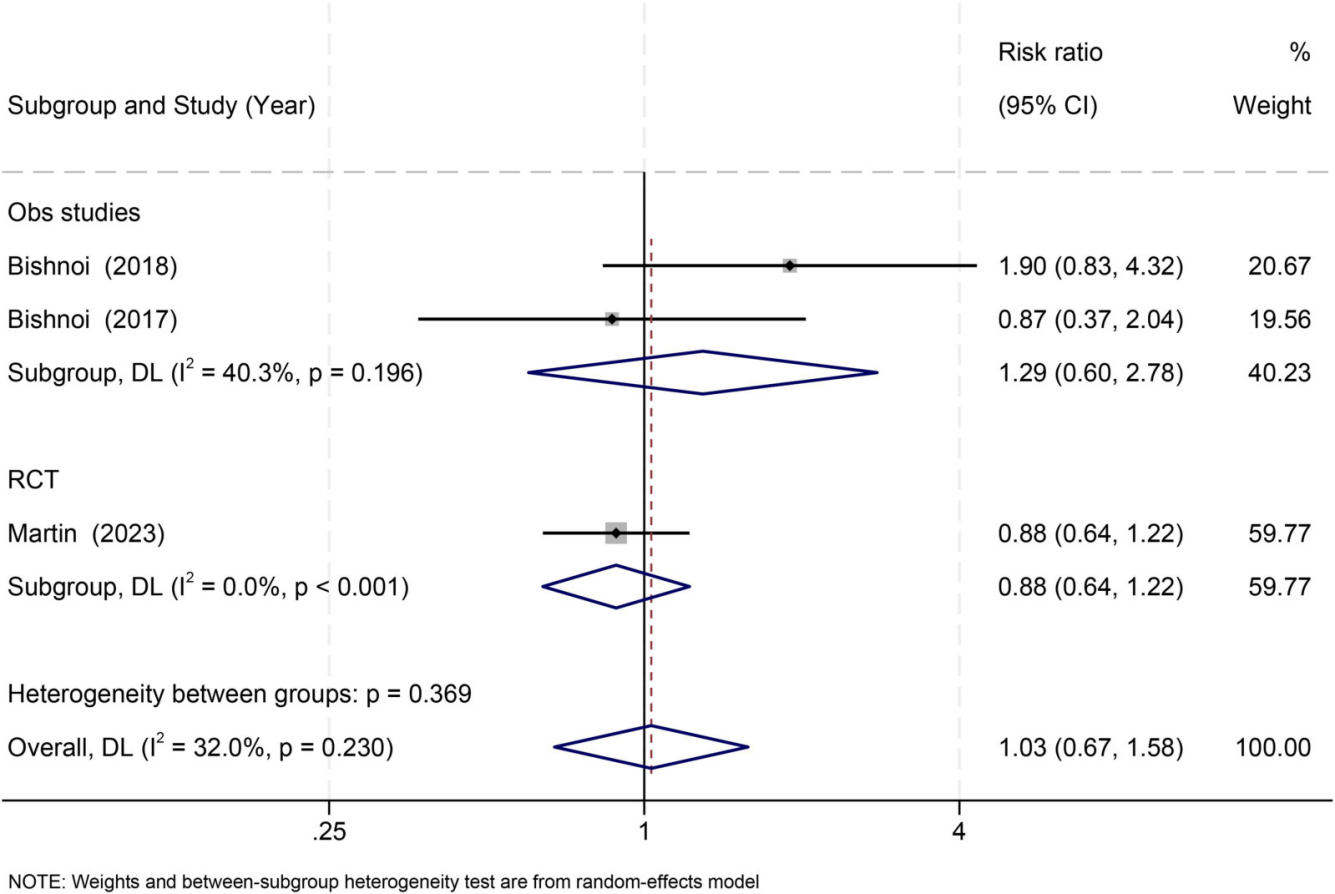
Effect of RBC storage duration on multiple organ dysfunction syndrome (MODS) in pediatric cardiac surgery: subgroup and overall estimates.

Three studies (32, 33, 36) reported MODS. No significant association was observed (RR = 1.03, 95% CI: 0.67–1.58; *I*^2^ = 32.0%). Subgroup results were consistent across observational studies (RR = 1.29) and the RCT (RR = 0.88) (*p* = 0.250) (Figure 5).

A ≤ 7-day threshold analysis produced similar null results, with no subgroup differences (*p* = 0.107) (Supplementary Figure S8).

Leave-one-out sensitivity analyses confirmed robustness (Supplementary Figure S9).

### 3.4 Meta-analysis of secondary outcomes

#### 3.4.1 Mechanical ventilation time

**Figure 6:**
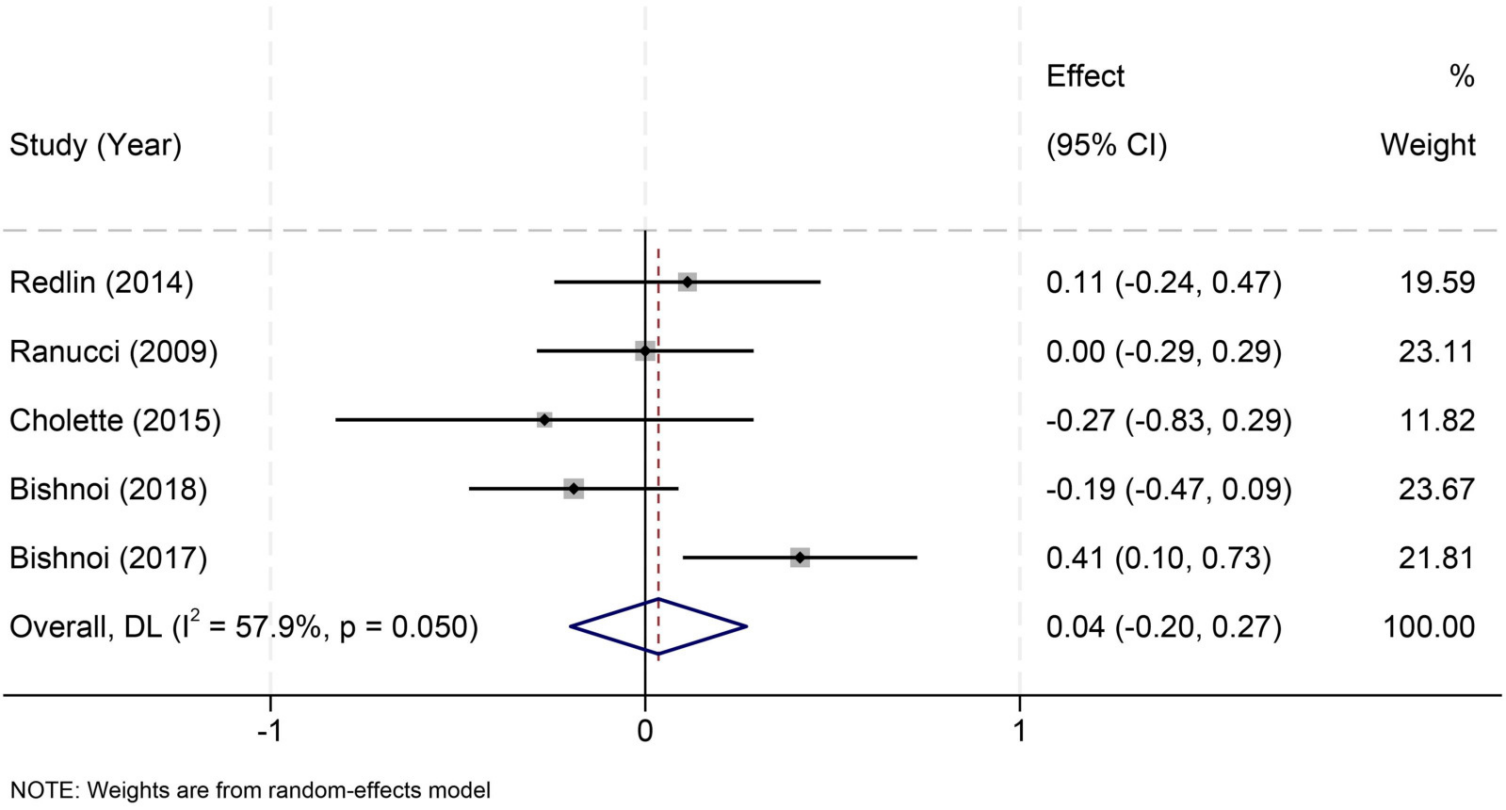
Effect of RBC storage duration on postoperative mechanical ventilation time in pediatric cardiac surgery.

Five studies (21–23, 32, 33) reported ventilation duration, with no significant difference between fresh and stored RBCs (MD = 0.04 h, 95% CI: −0.20 to 0.27; *I*^2^ = 57.9%) (Figure 6).

Threshold-standardized (≤7 vs. >7 days) and study-design subgroup analyses showed consistent null associations (Supplementary Figure S10).

Leave-one-out analyses, including exclusion of Ranucci 2009 (22), confirmed robustness (Supplementary Figure S11).

#### 3.4.2 ICU length of stay

**Figure 7:**
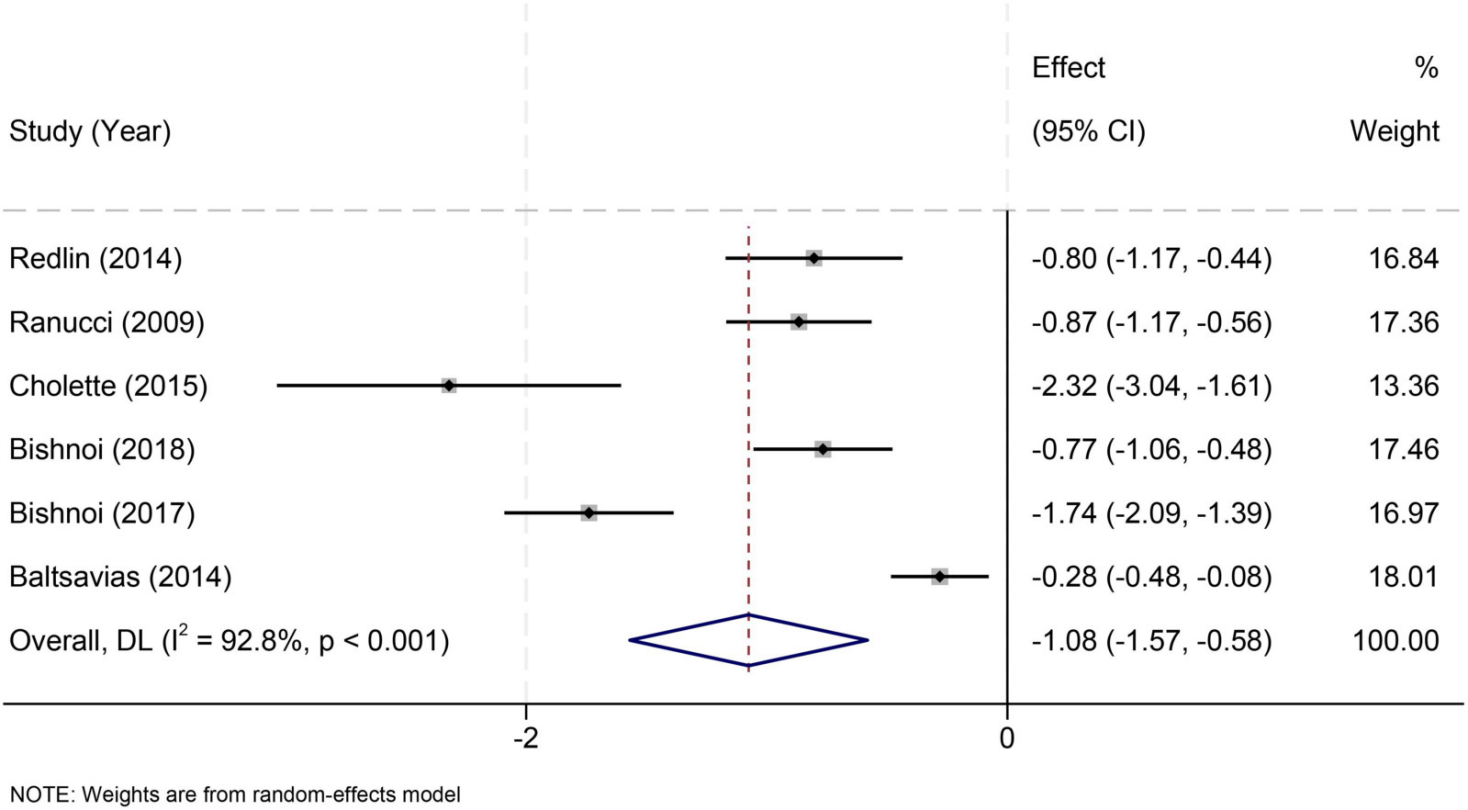
Effect of RBC storage duration on ICU length of stay in pediatric cardiac surgery.

Six studies (21–23, 30–33) reported ICU stay. Fresh RBCs were associated with shorter ICU stay (MD = –1.08 days, 95% CI: −1.57 to −0.58), although heterogeneity was high (*I*^2^ = 92.8%) (Figure 7).

Threshold-standardized ≤7-day analyses yielded similar findings, with no subgroup difference (*p* = 0.380) (Supplementary Figure S12).

Leave-one-out analyses confirmed that removal of Ranucci 2009 (22) did not meaningfully affect results (Supplementary Figure S13).

To further evaluate robustness under high heterogeneity, REML models were fitted, yielding estimates closely aligned with the DerSimonian–Laird model, without altering significance or direction of effect—supporting the stability of findings.

#### 3.4.3 Lactate levels during CPB

**Figure 8:**
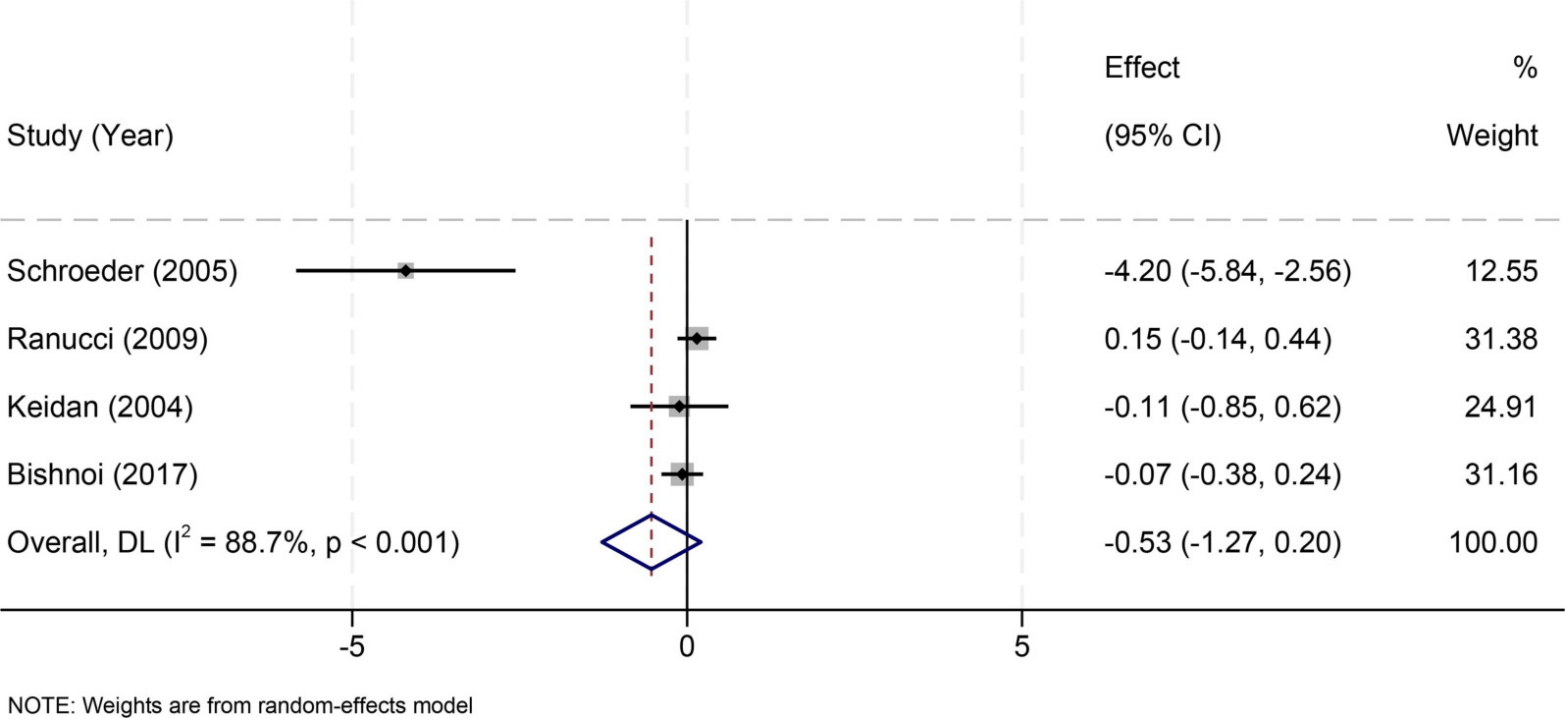
Effect of RBC storage duration on intraoperative lactate levels during cardiopulmonary bypass in pediatric cardiac surgery.

Four studies (32–35) assessed lactate levels during CPB. No significant difference was observed between fresh and older RBCs (MD = –0.53 mmol/L, 95% CI: −1.27 to 0.20; *I*^2^ = 88.7%) (Figure 8).

A ≤ 7-day subgroup analysis produced similar nonsignificant findings (Supplementary Figure S14).

Leave-one-out analyses—including exclusion of Ranucci 2009 (22)—showed no effect on pooled estimates (Supplementary Figure S15).

Given high heterogeneity, REML models were applied and produced results consistent with primary analyses, supporting robustness.

## 4 Discussion

In this systematic review and meta-analysis, we synthesized evidence from the past two decades regarding the impact of RBC storage duration on postoperative outcomes in pediatric cardiac surgery. Across all major clinical endpoints—including mortality, infection/sepsis, respiratory complications, and MODS—no statistically significant differences were found between fresher and older RBCs. Secondary outcomes, including mechanical ventilation duration and intraoperative lactate levels, similarly showed no meaningful differences. Leave-one-out sensitivity analyses applied to all primary and secondary outcomes demonstrated that no single study materially altered the pooled estimates, supporting the overall robustness of the findings across study designs, outcome types, and analytical approaches.

A key challenge in interpreting the evidence is the substantial variability in definitions of “fresh” RBCs, which ranged from ≤4 to ≤15 days across included studies. Such inconsistency inevitably introduced clinical heterogeneity and limited direct comparability. To address this, we implemented a threshold-standardized subgroup analysis using ≤7 days as a unified definition, selected because it represented a common definitional midpoint and allowed maximal study inclusion without compromising analytical comparability. Harmonizing storage thresholds improved interpretability and confirmed that variations in original definitions did not materially influence effect estimates.

One notable finding was a modest reduction in ICU length of stay (–1.08 days) in the fresher RBC group. However, this result arose predominantly from observational studies and exhibited substantial heterogeneity (*I*^2^ = 92.8%). To strengthen interpretability under such conditions, we additionally applied a REML random-effects model. The REML-derived estimates were highly consistent with those from the DerSimonian–Laird model, indicating that the observed direction and significance of effect were robust despite heterogeneity. Sensitivity analyses excluding the single study using an extremely strict ≤4-day definition [Ranucci 2009 (22)] further confirmed that threshold outliers did not exert undue influence.

Although stored RBCs undergo progressive “storage lesions”—including membrane instability, ATP and 2,3-DPG depletion, and release of pro-inflammatory mediators (7–12)—our findings did not identify a clinical advantage of fresher blood in pediatric CPB settings. Several mechanistic explanations may account for these neutral results. First, modern blood banking practices—such as universal leukoreduction, improved additive solutions, and optimized storage conditions—have substantially attenuated storage lesions compared with earlier decades, narrowing physiological differences between “fresh” and “older” units (11, 37). Second, transfusion practices often introduce confounding by indication: neonates or high-risk patients, who inherently experience poorer postoperative outcomes, are more likely to receive fresher units, biasing comparisons toward equivalence (38, 39). Third, the high-flow, hemodilutional environment of pediatric CPB rapidly dilutes transfused RBCs within the priming volume, reducing the effective load of storage-derived byproducts and mitigating their physiological impact (40, 41). Collectively, these considerations offer a biologically plausible explanation for the largely neutral findings observed.

While evidence from major adult RCTs such as RECESS and ABLE provides contextual reference (15, 16), direct extrapolation to pediatric CPB is limited by fundamental physiological differences. Neonates and infants possess immature organ systems, higher metabolic rates, and heightened inflammatory responses to CPB. These distinctions underscore the need to interpret adult data cautiously and highlight the importance of pediatric-focused evidence. Interestingly, two recent adult meta-analyses reported a potential increase in nosocomial infection risk with fresher RBC transfusion (42, 43), whereas observational studies in pediatric cardiac surgery have associated longer storage with increased infection risk (21, 22). Our findings fall between these perspectives, suggesting that storage duration alone may not be a dominant driver of infection risk in pediatric CPB, where multiple patient and management-related factors likely play more influential roles.

The neutral findings observed in this review align with current transfusion guidelines, which do not mandate preferential use of fresh RBCs in pediatric cardiac surgery (24). For institutions facing logistical limitations in blood inventory management, these results provide practical reassurance that using RBCs within approved storage periods for CPB priming is safe and feasible.

Although our meta-analysis integrates the most comprehensive available evidence, several limitations should be acknowledged. Most included studies were observational, limiting causal inference. Definitions of postoperative complications—especially respiratory complications—varied substantially across studies, potentially introducing misclassification bias. To address this, we categorized nonspecific descriptions (e.g., “pulmonary complications,” “respiratory events”) as mild and conducted a sensitivity analysis excluding such studies; notably, the effect estimate for severe respiratory complications remained unchanged, indicating that variability in outcome definitions did not drive the results. Furthermore, substantial variation existed across studies in CPB priming strategies, transfusion thresholds, postoperative management, and age distributions. Although we considered performing subgroup analyses based on age strata, CPB priming volume, or study era, the required stratified data were insufficiently reported to permit reliable analysis. These clinical inconsistencies likely contributed to residual heterogeneity and highlight the need for standardized reporting in future research.

To enhance methodological transparency, raw 2 × 2 event counts for all dichotomous outcomes were provided in Supplementary Table S1, as several original studies reported summary statistics without complete event distributions. Publication bias could not be formally assessed because none of the outcomes included the ≥10 studies required for reliable funnel plots or Egger's tests, and thus small-study effects cannot be excluded. Finally, although a meta-regression examining storage duration as a continuous moderator was considered, no outcome met the methodological minimum of ≥10 studies, and such analyses would likely produce unstable estimates.

In summary, despite extreme definitional variability and clinical heterogeneity across studies, threshold-standardized and sensitivity analyses demonstrated that effect estimates were generally stable. These findings suggest that RBC storage duration may exert less clinical influence in pediatric cardiac surgery than previously assumed. Future research—particularly multicenter RCTs with standardized reporting—remains essential to determine whether specific pediatric subgroups may derive benefit from fresher RBC transfusion.

## 5 Conclusion

This systematic review and meta-analysis synthesized current evidence and found no significant differences in major or secondary perioperative outcomes between fresher and longer-stored RBCs when used within the approved storage duration in pediatric cardiac surgery. These findings support the current practice guidelines that do not mandate the preferential use of fresher RBCs and provide valuable implications for both clinical decision-making and blood inventory management. Nevertheless, high-quality randomized controlled trials are still needed to further validate these conclusions and to develop more refined, evidence-based transfusion strategies tailored for pediatric populations.

## Data Availability

The original contributions presented in the study are included in the article/Supplementary Material, further inquiries can be directed to the corresponding authors.
