# Supplementary material for: The impact of red blood cell storage duration on clinical outcomes in pediatric cardiac surgery: a systematic review and meta-analysis
Source: Front Pediatr. 2025 Dec 16;13:1649610. doi: 10.3389/fped.2025.1649610 (PMC12748228; doi:10.3389/fped.2025.1649610)
Supplement: Supplementary file 1 [file Datasheet1.zip › Supplementary_Materials.docx]

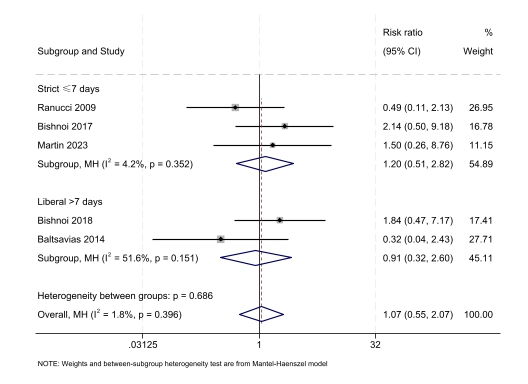


**Supplementary Figure S1.** Subgroup meta-analysis of postoperative mortality stratified by a standardized ≤7-day versus >7-day red blood cell storage threshold.


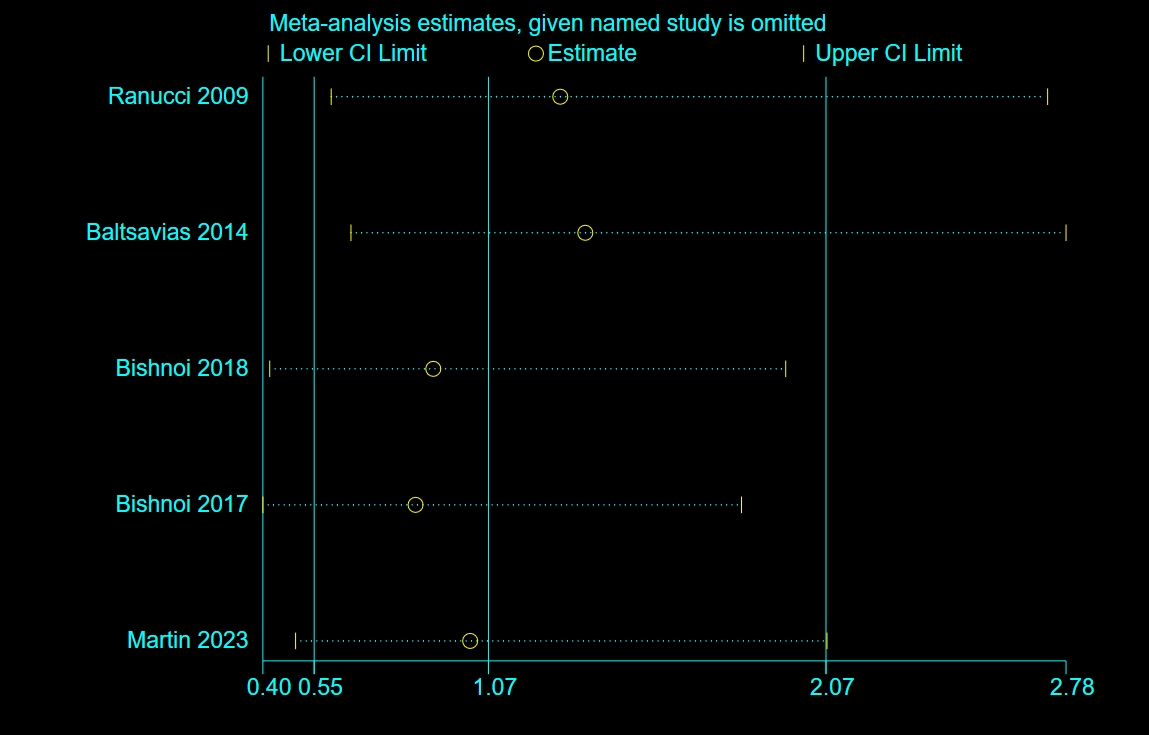


**Supplementary Figure S2. Sensitivity analysis for postoperative mortality**
This figure presents the leave-one-out sensitivity analysis for postoperative mortality. The pooled risk ratio and 95% confidence intervals are shown after sequential exclusion of each study. No single study significantly altered the overall effect estimate, indicating robust results.


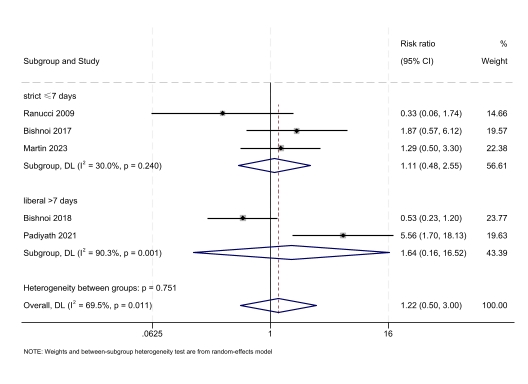


**Supplementary Figure S3.** Subgroup Analysis of Sepsis Stratified by a Standardized ≤7-Day Versus >7-Day RBC Storage Threshold.

**Supplementary Figure S4.** Subgroup analysis of postoperative infection stratified by a standardized ≤7-day versus >7-day RBC storage threshold


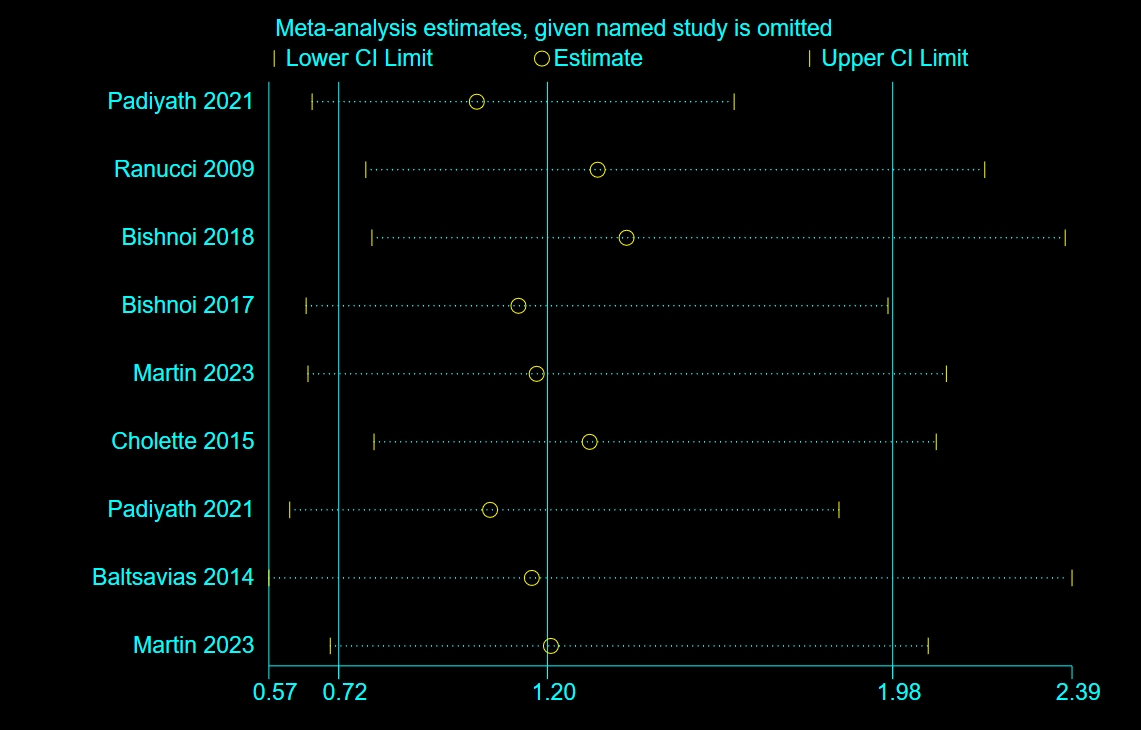


**Supplementary Figure S5. Sensitivity analysis for postoperative infection and sepsis**
This figure presents the leave-one-out sensitivity analysis for postoperative infection and sepsis. Effect estimates remain stable across exclusions, suggesting that the meta-analysis result is not driven by any single study.


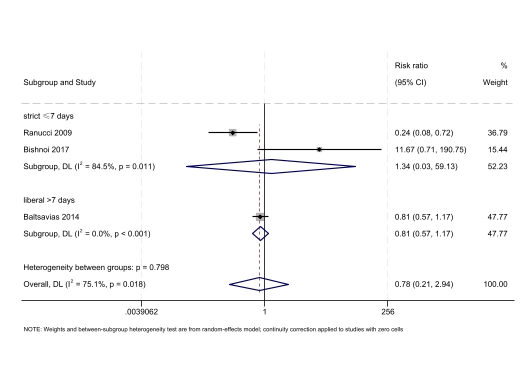


**Supplementary Figure S6.** Subgroup analysis of severe respiratory complications stratified by a standardized ≤7-day versus >7-day RBC storage threshold.

**
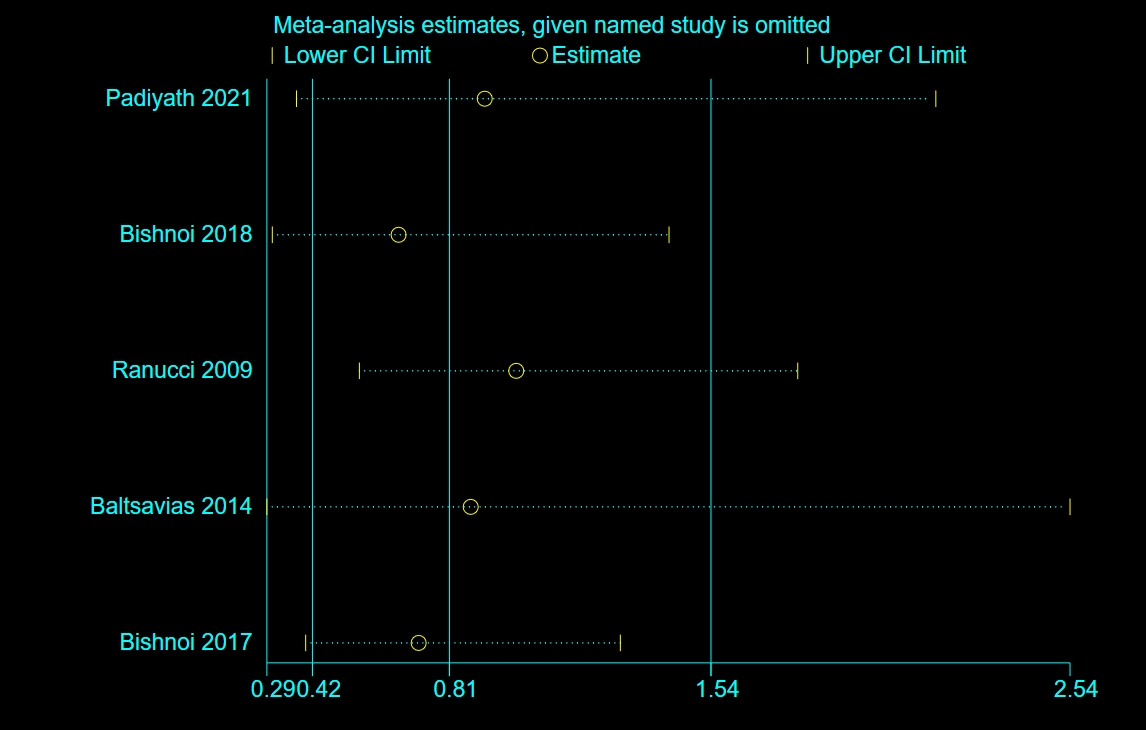
**

**Supplementary Figure S7. Sensitivity analysis for respiratory complications**
This figure displays the leave-one-out sensitivity analysis for respiratory complications. Consistent effect estimates across study exclusions confirm the reliability of the pooled results.

**Supplementary Figure S8.** Subgroup analysis of MODS stratified by a standardized ≤7-day versus >7-day RBC storage threshold.


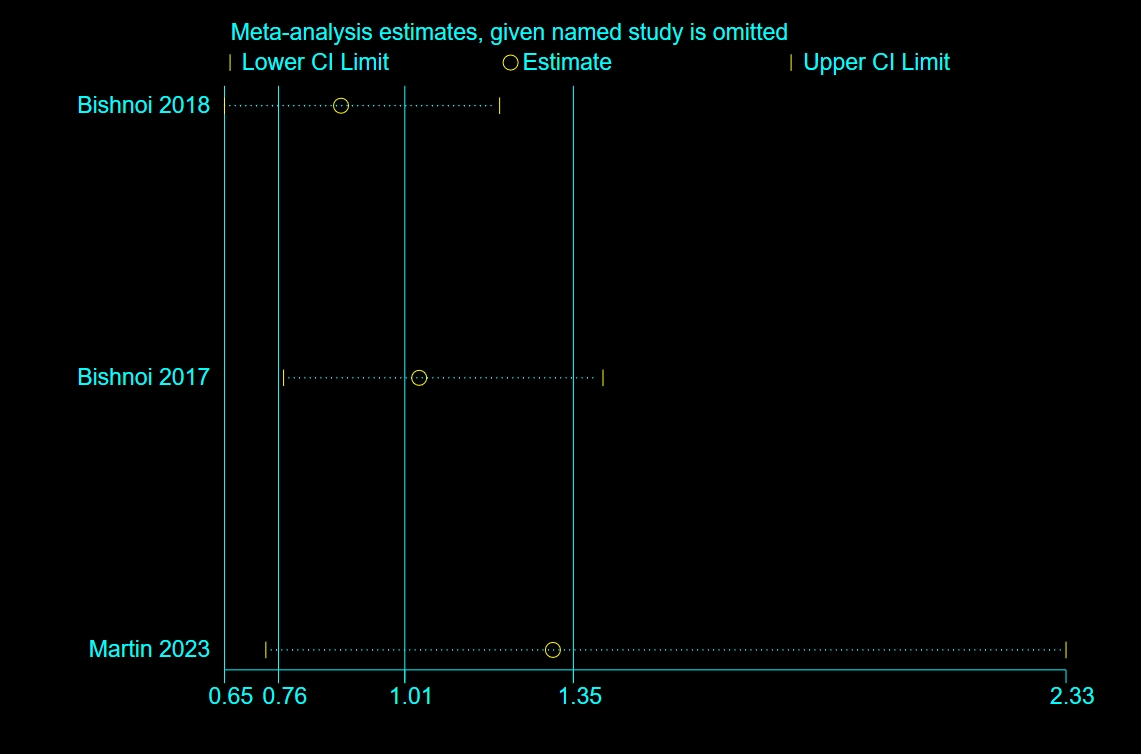


#### Supplementary Figure S9. Sensitivity analysis for multiple organ dysfunction syndrome (MODS) This figure illustrates the leave-one-out sensitivity analysis for MODS. Removal of individual studies did not materially change the overall effect, supporting the robustness of the findings.

Supplementary Figure S10. Subgroup analysis of mechanical ventilation duration stratified by a standardized ≤7-day versus >7-day RBC storage threshold.


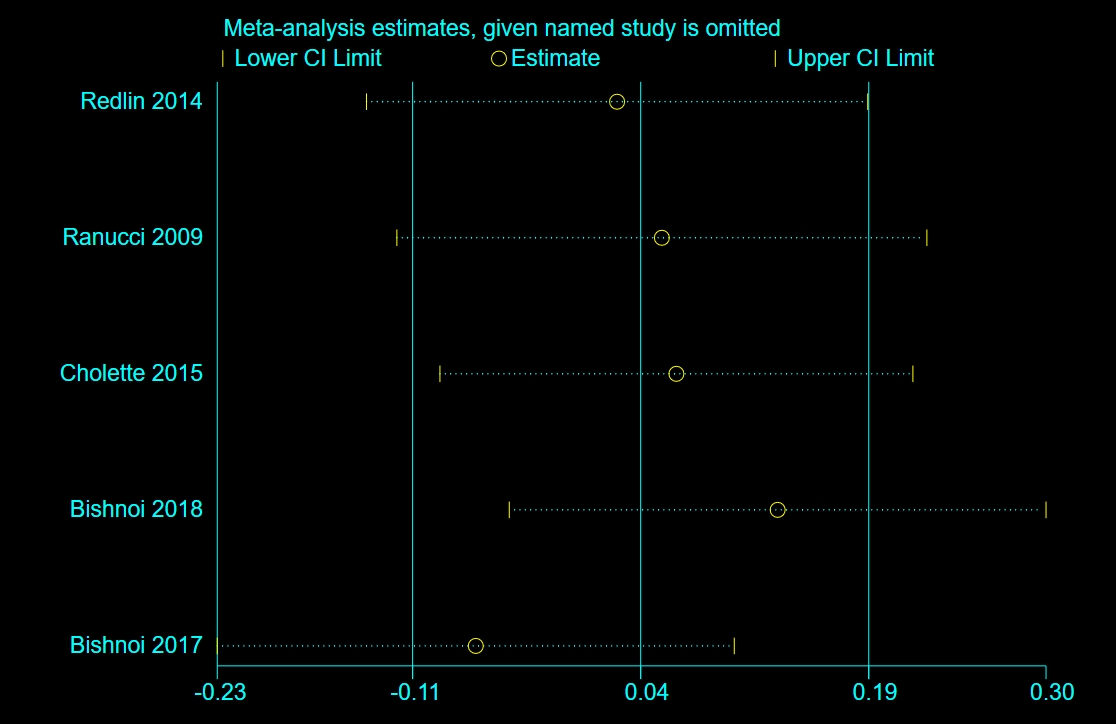


Supplementary Figure S11.Sensitivity analysis of mechanical ventilation duration

Supplementary Figure S12. Subgroup analysis of ICU length of stay stratified by a standardized ≤7-day versus >7-day RBC storage threshold.


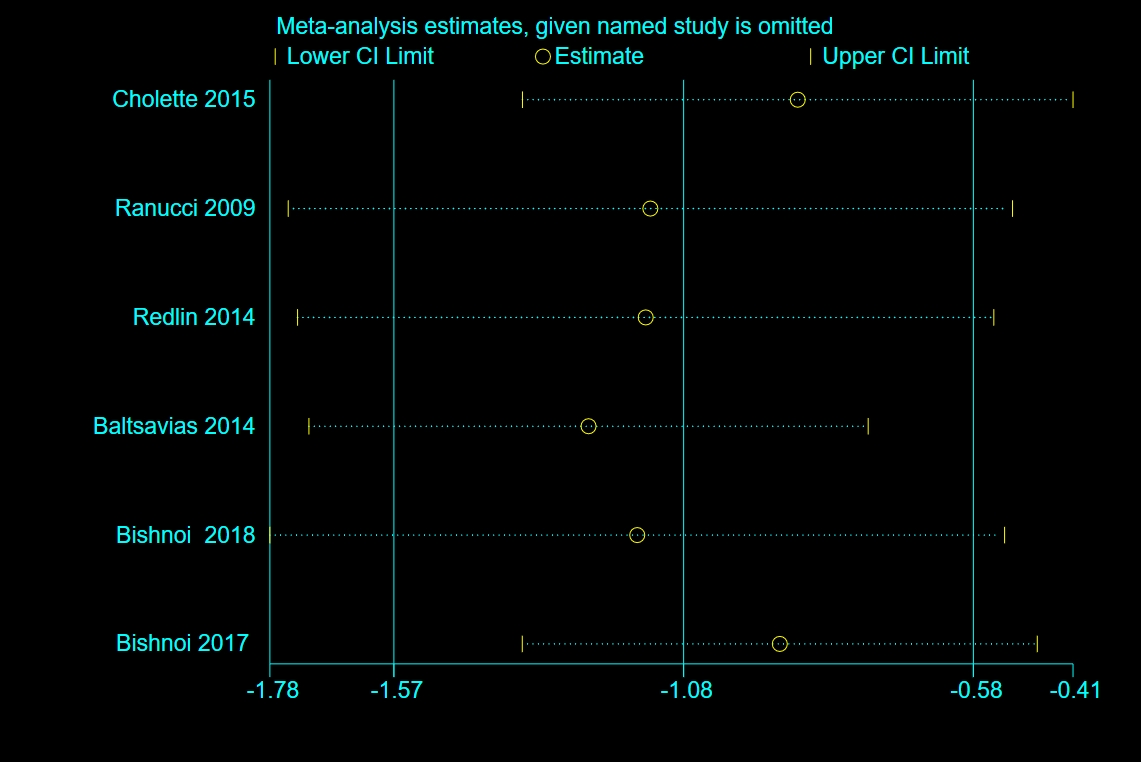


Supplementary Figure S13. Sensitivity analysis of ICU length of stay


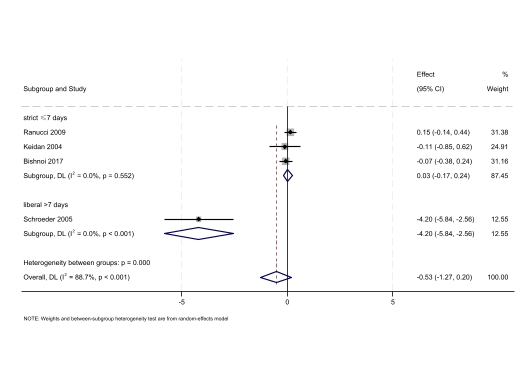


Supplementary Figure S14. Subgroup analysis of lactate levels during CPB stratified by a standardized ≤7-day versus >7-day RBC storage threshold.


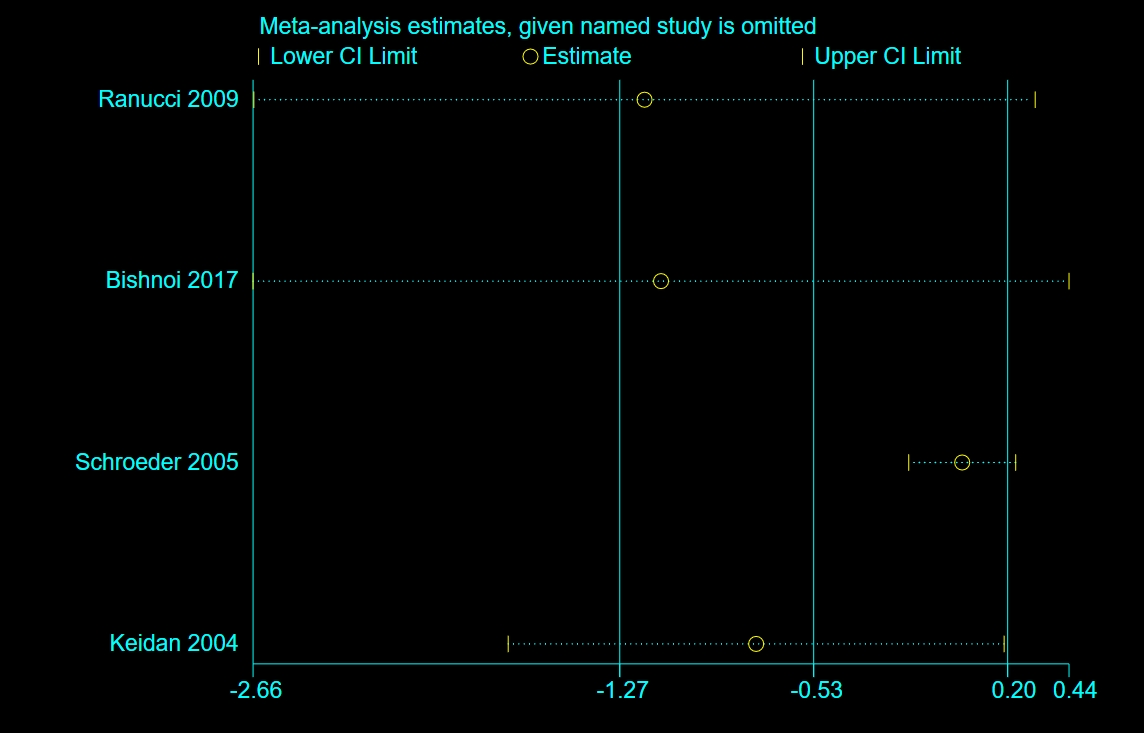


Supplementary Figure S15. Sensitivity analysis of lactate levels during CPB.
