# Supplementary material for: The impact of red blood cell storage duration on clinical outcomes in pediatric cardiac surgery: a systematic review and meta-analysis
Source: Front Pediatr. 2025 Dec 16;13:1649610. doi: 10.3389/fped.2025.1649610 (PMC12748228; doi:10.3389/fped.2025.1649610)
Supplement: Supplementary file 1 [file Datasheet1.zip › Supplementary_Methods..docx]

Supplementary Table S1. Raw 2×2 event counts (a, b, c, d) for dichotomous outcomes by study

| Outcome Category | Study  (Year) | Fresh Events  (a) | Fresh Non-Events  (b) | Stored Events  (c) | Stored Non-Events  (d) |
| --- | --- | --- | --- | --- | --- |
| Mortality | Ranucci 2009 | 3 | 113 | 4 | 72 |
|  | Bishnoi 2018 | 6 | 97 | 3 | 92 |
|  | Bishnoi 2017 | 13 | 148 | 2 | 51 |
|  | Baltsavias 2014 | 1 | 117 | 12 | 440 |
|  | Martin 2023 | 3 | 86 | 2 | 87 |
| Sepsis | Ranucci 2009 | 2 | 114 | 4 | 72 |
|  | Bishnoi 2018 | 8 | 95 | 14 | 81 |
|  | Bishnoi 2017 | 17 | 144 | 3 | 50 |
|  | Padiyath 2021 | 8 | 114 | 4 | 335 |
|  | Martin 2023 | 9 | 80 | 7 | 82 |
| Infection | Cholette 2015 | 0 | 33 | 4 | 16 |
|  | Padiyath 2021 | 10 | 112 | 12 | 327 |
|  | Martin 2023 | 2 | 87 | 2 | 87 |
|  | Baltsavias 2014 | 61 | 57 | 210 | 242 |
| MODS (Multiple Organ Dysfunction Syndrome) | Bishnoi 2018 | 14 | 81 | 8 | 95 |
|  | Bishnoi 2017 | 6 | 47 | 21 | 140 |
|  | Martin 2023 | 38 | 51 | 43 | 46 |
| Mild Respiratory Complications | Bishnoi 2018 | 13 | 82 | 9 | 94 |
|  | Padiyath 2021 | 15 | 324 | 9 | 113 |
| Severe Respiratory Complications | Ranucci 2009 | 4 | 112 | 11 | 65 |
|  | Bishnoi 2017 | 17 | 144 | 0 | 53 |
|  | Baltsavias 2014 | 27 | 91 | 127 | 325 |
| Liver Complications | Bishnoi 2018 | 3 | 100 | 17 | 78 |
|  | Bishnoi 2017 | 7 | 154 | 10 | 43 |
| Renal Complications | Bishnoi 2018 | 8 | 95 | 8 | 87 |
|  | Bishnoi 2017 | 13 | 148 | 4 | 49 |

# PRISMA 2020 Checklist

This PRISMA 2020 checklist was completed for the manuscript titled 'The Impact of Red Blood Cell Storage Duration on Clinical Outcomes in Pediatric Cardiac Surgery: A Systematic Review and Meta-Analysis'. This checklist ensures transparent and comprehensive reporting in accordance with the PRISMA 2020 statement.

| Section & Topic | Checklist Item | Reported Location in Manuscript |
| --- | --- | --- |
| TITLE | Identify the report as a systematic review/meta-analysis. | Title page |
| ABSTRACT | Structured summary with background, objectives, methods, results, conclusion. | Abstract section |
| INTRODUCTION | Describe the rationale for the review. | Introduction, paragraph 1–5 |
| INTRODUCTION | Explicit statement of the objectives. | Introduction, final paragraph |
| METHODS | Specify inclusion and exclusion criteria. | Methods 2.3 |
| METHODS | Describe all databases and search dates. | Methods 2.2 |
| METHODS | Present full search strategy for at least one database. | Supplementary Table 1 |
| METHODS | Methods used to screen and include studies. | Methods 2.3 |
| METHODS | Methods of data extraction and missing data handling. | Methods 2.4 |
| METHODS | Define all outcomes and data collected. | Methods 2.4 |
| METHODS | Risk of bias assessment methods. | Methods 2.5 |
| METHODS | Effect measures used for outcomes. | Methods 2.6 |
| METHODS | Meta-analysis model, heterogeneity assessment, subgroup & sensitivity analysis. | Methods 2.6 |
| METHODS | Assessment of reporting bias (if done). | Not performed |
| METHODS | Certainty assessment (e.g., GRADE). | Methods 2.7 |
| RESULTS | Number of studies screened/included; exclusion reasons. | Results 3.1; Figure 1 |
| RESULTS | Characteristics of included studies. | Results 3.1; Table 1 |
| RESULTS | Risk of bias results for each study. | Results 3.2; Table 2–3 |
| RESULTS | Summary statistics for each outcome and study. | Results 3.3–3.5; Figures 2–8 |
| RESULTS | Meta-analysis results with CIs and heterogeneity. | Results 3.3–3.5 |
| RESULTS | Results of reporting bias assessment (if done). | Not performed |
| RESULTS | Certainty of evidence summary. | Methods 2.7; briefly in Discussion |
| DISCUSSION | Summary of main findings and strength of evidence. | Discussion, paragraph 1–2 |
| DISCUSSION | Limitations of included evidence. | Discussion, paragraph 5 |
| DISCUSSION | Interpretation of results in context of other evidence. | Discussion, paragraph 3–4 |
| DISCUSSION | Implications for practice, policy, future research. | Discussion, paragraph 5–6 |
| OTHER | Registration and protocol info. | Methods 2.1; PROSPERO No. CRD420251015198 |
| OTHER | Sources of funding/support. | Funding section |
| OTHER | Authors’ conflicts of interest. | Conflicts of Interest section |
| OTHER | Availability of data/code. | Not stated; can be added if needed |

# Database Search Strategies:

| Search strategy: ("Pediatrics"[Mesh] OR "Child"[Mesh] OR "Infant"[Mesh] OR "pediatric cardiac surgery" OR "congenital heart disease" OR "children" OR "infants") AND ("Erythrocytes"[Mesh] OR "Blood Transfusion"[Mesh] OR "red blood cell storage" OR "RBC storage" OR "CPB priming" OR "cardiopulmonary bypass") AND ("clinical outcomes" OR "mortality" OR "infection rate" OR "postoperative complications") |
| --- |

Table 1. Example of Boolean logic operators used to identify studies on red blood cell storage duration in pediatric cardiac surgery (PubMed search strategy).

| ('pediatrics'/exp OR 'child'/exp OR 'infant'/exp OR 'pediatric cardiac surgery' OR 'congenital heart disease' OR 'children' OR 'infants') AND  ('erythrocyte'/exp OR 'blood transfusion'/exp OR 'red blood cell storage' OR 'RBC storage' OR 'CPB priming' OR 'cardiopulmonary bypass') AND  ('clinical outcome'/exp OR 'mortality'/exp OR 'infection rate' OR 'postoperative complication') |
| --- |

Table 2. Example of Boolean logic operators used to identify studies on red blood cell storage duration in pediatric cardiac surgery (EMBASE search strategy).

| ([mh "Pediatrics"] OR [mh "Child"] OR [mh "Infant"] OR "pediatric cardiac surgery" OR "congenital heart disease" OR "children" OR "infants") AND  ([mh "Erythrocytes"] OR [mh "Blood Transfusion"] OR "red blood cell storage" OR "RBC storage" OR "CPB priming" OR "cardiopulmonary bypass") AND  ("clinical outcomes" OR [mh "Mortality"] OR "infection rate" OR [mh "Postoperative Complications"]) |
| --- |

Table 3. Example of Boolean logic operators used to identify studies on red blood cell storage duration in pediatric cardiac surgery (Cochrane Library search strategy).

| TS=("pediatric cardiac surgery" OR "congenital heart disease" OR "children" OR "infants") AND  TS=("red blood cell storage" OR "RBC storage" OR "blood transfusion" OR "CPB priming" OR "cardiopulmonary bypass") AND  TS=("clinical outcomes" OR "mortality" OR "infection rate" OR "postoperative complications") |
| --- |

Table 4. Example of Boolean logic operators used to identify studies on red blood cell storage duration in pediatric cardiac surgery (Web of Science search strategy).
